# Age-related cataract without surgery is related to exacerbated depression symptoms: a cross-sectional study of Chinese adults from Anhui, China

**DOI:** 10.3389/fmed.2024.1483709

**Published:** 2024-10-31

**Authors:** Tao Wang, Hexia Li, Quangang Cao

**Affiliations:** ^1^Department of Ophthalmology, The Lu’an Hospital Affiliated to Anhui Medical University, Lu’an, Anhui, China; ^2^Department of Ophthalmology, The Lu’ an People’s Hospital, Lu’an, Anhui, China; ^3^Graduate School of Bengbu Medical University, Bengbu, Anhui, China

**Keywords:** age-related cataract, depression, surgery, elderly, cross-sectional study

## Abstract

**Objective:**

The present study sought to evaluate the relationship between age-related cataracts, a prevalent ocular condition among the elderly, and the occurrence of depressive symptoms within a cohort of Chinese adults residing in Anhui, China.

**Methods:**

A survey involving 252 Chinese individuals aged 65 years and older was conducted at Lu’an People’s Hospital. Depressive symptoms were assessed using the Hamilton Depression Scale (HAMD) consisting of 17 items, while age-related cataracts were clinically classified according to the Lens Opacities Classification System (LOCS) III. Depressive symptoms were identified by a HAMD score exceeding 7. Logistic regression analyses were employed to determine the odds ratios (OR) and 95% confidence intervals (CI) pertaining to the association between age-related cataracts and depressive symptoms.

**Results:**

Cataract patients aged 65 years and older had significantly higher scores of depressive symptoms than those under 65 years (mean scores of 8.17 ± 3.26 versus 5.18 ± 1.86, *p* < 0.001). In addition, patients aged 65 years and above exhibited a diminished quality of life relative to patients aged under 65 years. The findings indicated that adults experiencing depressive symptoms reported lower monthly incomes (*p* = 0.036), lower educational attainment (*p* = 0.044), and living alone (*p* = 0.007). Furthermore, fewer elderly patients with depressive symptoms received surgical treatment (15 patients) than those without depressive symptoms (61 patients), with a significant difference (*p* = 0.011). Multivariate analysis revealed that the presence of depressive symptoms was significantly correlated with a lack of formal education (*p* = 0.048), reduced income (*p* = 0.003), solitary living arrangements (*p* = 0.025), and the presence of cataracts without surgical intervention (*p* = 0.007).

**Conclusion:**

These findings suggested a significant association between age-related cataracts and depressive symptoms among older adults. Specifically, cataracts that remain untreated surgically were notably linked to depression in elderly patients. It is recommended that initiatives and resources be directed toward surgical treatment programs for cataracts in individuals exhibiting depressive symptoms.

## Introduction

Age-related cataract is recognized as the primary cause of mild to moderate visual impairment globally (1, 2). As life expectancy increases and the population ages, the prevalence and impact of age-related cataracts are anticipated to rise, thereby presenting a significant public health challenge on a global scale. Economic evaluations conducted in the United States consistently indicated that the medical expenses associated with age-related cataracts substantially surpass those related to other major ocular conditions (3). A recent investigation revealed considerable variability in average treatment costs per patient across various conditions, including refractive error correction (ranging from $12 to $201 per patient per procedure), cataract surgery (ranging from $54 to $3,654 per patient per procedure), and glaucoma (ranging from $351 to $1,354 per patient per procedure) (4). Consequently, cataracts impose significant economic burdens on individuals, communities, and nations.

Depression is a long-lasting and often recurring mental health disorder that is common among older adults, but its connection to cataracts is not well understood. Previous studies have shown that depression is frequently encountered in eye care settings, often going unnoticed or untreated (5–7). Many investigations have looked into the link between visual impairment and depression, but the findings have been inconsistent. For example, a study of 339 socially vulnerable adults over 50 in Armenia revealed that those with visual impairment had a significantly higher risk of depression than those without visual impairment, even after accounting for other factors (8). In contrast, a European study found that older adults with visual impairments had a notably higher prevalence of major depressive disorder and anxiety than those with normal vision (9). However, other studies have shown no significant relationship. A population-based study in the United States involving 2,520 individuals aged 65 to 84 years found no link between visual acuity or its changes and the development of depressive symptoms (10). In addition, a study of younger United States adults aged 20 to 39 found no correlation between visual acuity and depressive disorders after adjusting for various factors (11). While some studies suggest that cataract surgery may lead to improvements in depressive symptoms (12–15), there is still limited understanding of how cataracts relate to depression, especially in the Chinese population.

Investigating the risk factors associated with depression within the Chinese population holds significant public health relevance, given that the Chinese represent the largest ethnic group globally and may experience a substantial prevalence of depression. Furthermore, mental health issues are often accompanied by social stigma in Chinese cultural contexts. Research that examines the possible link between cataracts and depressive symptoms in the general population could guide clinical strategies for cataract management and impact public health policies. This study sought to assess the relationship between age-related cataracts and depressive symptoms in a community-based group of older Chinese adults aged 65 years and older. In addition, we aimed to assess whether the association between cataracts and depressive symptoms can be attributed to visual problems without immediate surgical intervention.

## Methods

### Study population

The research was a cross-sectional survey carried out in Lu’an, China, with the objective of assessing the patterns, predictors, and prevalence of common health outcomes among elderly individuals aged 65 years and older in eastern China. The methodology of the study has been detailed in a previous study (15). A total of 252 participants aged 65 years or older and 569 individuals under the age of 65 were recruited from our hospital for this investigation. A cataract diagnosis was confirmed through at least one inpatient or two outpatient assessments by an ophthalmologist, using the ICD-9-CM diagnosis code 366. The index date refers to the date when the cataract was first diagnosed. Participants in the cataract group were then divided into those who underwent surgery and those who did not undergo surgery to evaluate the impact of cataract surgery on depression risk. This research was approved by the Institutional Review Board of Lu′an People’s Hospital (2023LL027). All participants provided written informed consent during the recruitment phase of the study.

### Questionnaires

Cataract grading was performed using a slit-lamp examination (model SL-1E; Topcon) on both eyes of each participant in the study. This evaluation involved a clinical assessment of lens opacity based on the Lens Opacities Classification System (LOCS) III (16). The LOCS III system includes the assessment of nuclear opalescence (NO), cortical cataract (C), and posterior subcapsular cataract (PSC). A LOCS III score of 4.0 or higher for NO was considered indicative of a significant nuclear cataract, while scores of 2.0 or higher for C and PSC were regarded as significant, respectively (13). The presence of any cataract was defined as having at least one subtype in one eye.

To assess depressive symptoms, the 17-item Hamilton Depression Scale (HAMD) was used to gage the frequency of symptoms reported by participants over the last 2 weeks (17). The HAMD consists of 17 items divided into five categories. This scale employs a 5-point Likert scale, where 0 indicates no symptoms and 4 signifies extremely severe symptoms. A total HAMD score above 7 suggests the presence of depression (18). The scale has been validated for use in the general Chinese population and was translated into Chinese by Wang et al. for depression screening (19).

Furthermore, the Hamilton Anxiety Scale (HAMA) was used to assess patients’ mental states, featuring two subscales: psychic anxiety and somatic anxiety. The HAMA consists of 14 items, each rated on a 5-point Likert scale from 0 (no symptom) to 4 (extremely severe symptoms).

Quality of life was measured using the 36-item Short Form Survey (SF-36), a self-administered tool designed to evaluate health-related quality of life (QoL) across eight areas: physical functioning (PF), role limitations due to physical issues (RP), and others (20). Scores for each dimension ranged from 0 to 100, with higher scores indicating a better QoL. The SF-36 has been validated in a Chinese context by Ren et al. (21).

### Assessment of covariates

The evaluation of factors related to visual acuity was performed using a Snellen vision chart with tumbling-E optotypes (Precision Vision, La Salle, IL). Measurements were taken for each eye separately under lighting conditions of approximately 500 lux from a distance of 4 m, with participants wearing their prescribed vision aids, such as glasses or contact lenses, if necessary. The light levels in the examination room during the visual acuity test were measured using a light meter. Furthermore, a risk factor questionnaire was verbally administered by trained research assistants, and the information included participants’ socioeconomic status, lifestyle habits, medical history, and medication use. Diabetes mellitus was defined as either fasting glucose levels or doctors’ diagnosis of diabetes along with the use of diabetes medications. Hypertension was identified based on the WHO diagnostic criteria for hypertension and the patients’ medical history, including the use of antihypertensive medications.

### Statistical analysis

Data analyses were conducted using SPSS 16.0 (SPSS Inc., Chicago, IL). Participants who had cataract surgery in both eyes were not included in the analysis as their vision was severely affected. Binary logistic regression models were used to calculate odds ratios (ORs) and 95% confidence intervals (CIs) to examine the relationship between age-related cataracts and depressive symptoms. In the multivariate analysis, the model included only age, gender, cataract status, and other variables that showed significant differences in univariate comparisons (*p* < 0.05). The interactions between age-related cataracts and other variables related to depressive symptoms were assessed using an OR value. A *p*-value of less than 0.05 is indicative of statistical significance.

## Results

### Demographic characteristic between cataract patients with ≥65 years and < 65 years

A total of 252 individuals aged 65 years and older and 569 individuals under 65 years participated in this study. The mean age of cataract patients aged 65 years and older was significantly greater than that of patients under 65 years (70.58 ± 4.16 vs. 45.67 ± 7.26, *p* < 0.001). No significant differences were observed between the two groups in terms of gender distribution or complications (see Table 1).

**Table 1:** The demographic characteristics, mental health, and quality of life between cataracts patients with different age.

	Cataracts with aged ≥65 years (*n* = 252)	Cataracts with aged <65 years (*n* = 569)	*t*/*χ*2	*p*
Age (years, x ± s)	70.58 ± 4.16	45.67 ± 7.26	10.268	**<0.001**
Gender (*n*)				
Men	127	282	0.049	0.825
Women	125	287		
Complications				
Hypertension (*n*)	51	97	1.203	0.273
Diabetes mellitus (*n*)	59	102	3.335	0.068
Cancers	5	11	0.252	0.616
Mental health				
HAMA	5.18 ± 2.13	5.23 ± 1.99	1.258	0.369
HAMD	8.17 ± 3.26	5.18 ± 1.86	5.287	**<0.001**
Quality of life (SF-36)				
PF	65.21 ± 14.38	66.37 ± 15.27	−1.018	0.398
RP	54.50 ± 11.53	62.16 ± 14.38	−4.366	**0.004**
BP	57.84 ± 18.21	65.44 ± 11.26	−5.012	**0.002**
GH	55.89 ± 14.41	65.58 ± 13.57	−7.896	**<0.001**
VT	58.26 ± 11.33	65.28 ± 17.19	−5.216	**0.001**
SF	59.46 ± 17.89	61.88 ± 13.16	−1.287	0.321
RE	52.87 ± 14.20	63.17 ± 14.21	−8.012	**<0.001**
MH	65.39 ± 11.69	64.36 ± 14.58	0.578	0.601

PF, physical functioning; RP, physical problems; BP, bodily pain; GH, general health; VT, vitality; SF, social functioning; RE, role limitations due to emotional problems; and MH, mental health. Bold values mean *p* < 0.05.

### Mental health and quality of life between cataract patients with ≥65 years and < 65 years

**Figure 1:**
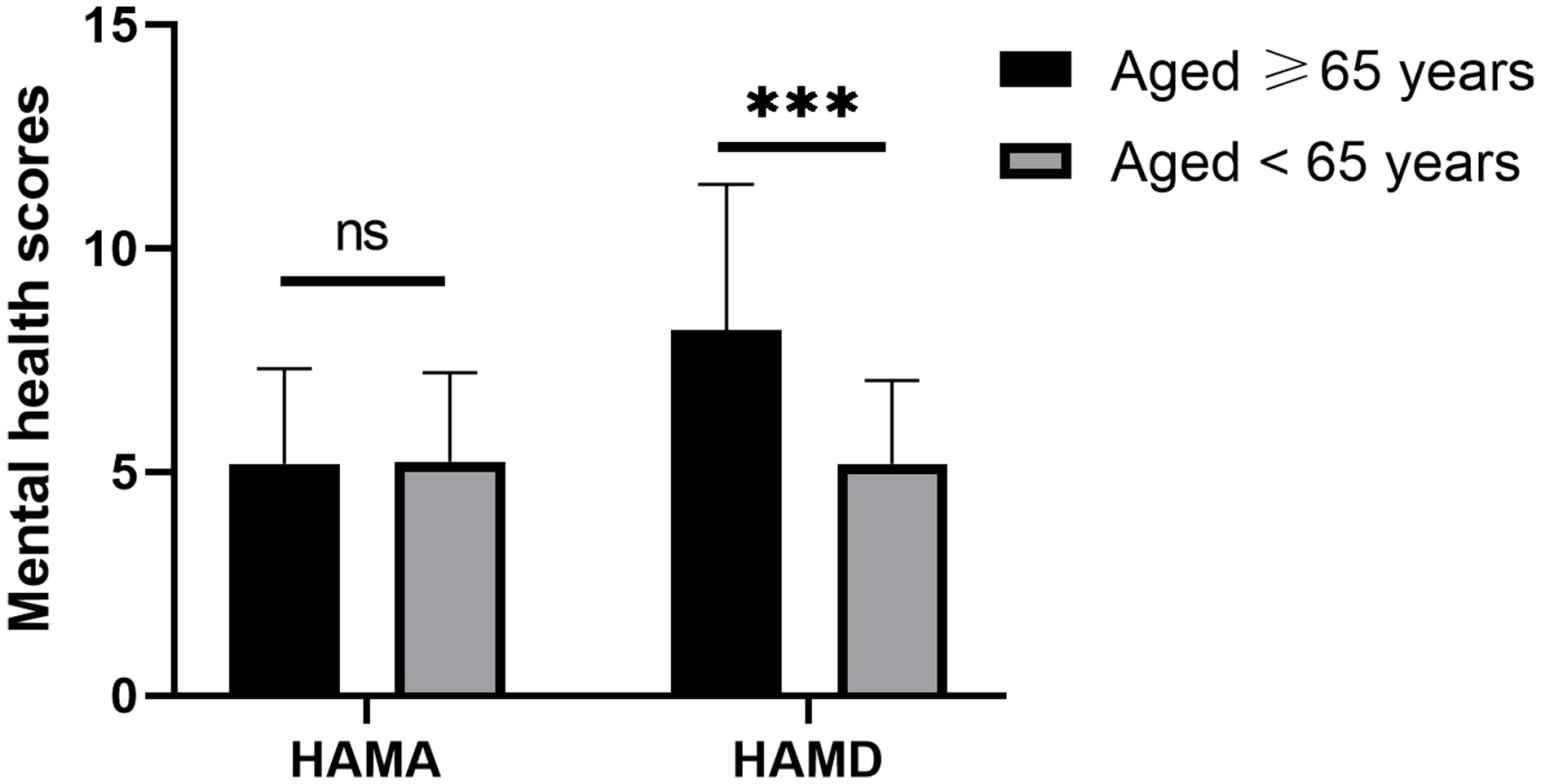
HAMA and HAMD between cataract patients of different ages. *** *p* < 0.001.

The analysis revealed no significant difference in the Hamilton Anxiety Rating Scale (HAMA) scores between the two groups (*t* = 1.258, *p* = 0.369). However, it is noteworthy that cataract patients aged 65 years and older had significantly higher Hamilton Depression Rating Scale (HAMD) scores (8.17 ± 3.26 vs. 5.18 ± 1.86, *p* < 0.001) than those under 65 years (Figure 1). In addition, cataract patients aged 65 years and older had lower scores in the following domains: role physical (RP) (54.50 ± 11.53 vs. 62.16 ± 14.38, *p* = 0.004), bodily pain (BP) (57.84 ± 18.21 vs. 65.44 ± 11.26, *p* = 0.002), general health (GH) (55.89 ± 14.41 vs. 65.58 ± 13.57, *p* < 0.001), vitality (VT) (58.26 ± 11.33 vs. 65.28 ± 17.19, *p* = 0.001), and role emotional (RE) (52.87 ± 14.20 vs. 63.17 ± 14.21, *p* < 0.001) when compared to their younger counterparts. These findings are summarized in Table 1.

### Basic information between cataract patients with depression and without depression symptoms

Table 2 presents a summary of the characteristics of the study participants categorized by the presence of depressive symptoms, as assessed by the Hamilton Depression Rating Scale (HAMD). The findings indicated that adults exhibiting depressive symptoms reported lower monthly income (*p* = 0.036), lower levels of education (*p* = 0.044), and living alone (*p* = 0.007). Furthermore, a smaller number of elderly patients with depressive symptoms had undergone surgery (15 vs. 61, *p* = 0.011) than those without depressive symptoms.

**Table 2:** Characteristics of study participants by the status of depressive symptoms.

	Elderly patients with depression (*n* = 78)	Elderly patients without depression (*n* = 174)	*t*/*χ*2	*p*
Age (years, x ± s)	70.13 ± 4.28	71.02 ± 4.16	1.268	0.259
Gender (*n*)				
Men (*n*)	46	85	2.211	0.137
Women (*n*)	32	89		
Surgery (*n*)				
Cataract with surgery (*n*)	15	61	6.455	**0.011**
Cataract without surgery (*n*)	63	113		
Individual monthly income (> 1,000 Yuan) (*n*)	24	78	4.418	**0.036**
No formal education (*n*)	42	70	4.044	**0.044**
Living alone (*n*)	41	60	7.332	**0.007**
Smoking history (*n*)	26	52	0.300	0.584
Alcohol intake (*n*)	20	38	0.439	0.507
Tea consumption (*n*)	19	44	0.025	0.875
Hypertension (*n*)	28	43	3.329	0.068
Diabetes mellitus (*n*)	21	38	0.766	0.378

Bold values mean *p* < 0.05.

### Multivariate logistic regression analysis

The relationships between depressive symptoms, cataract surgery, and various risk factors were analyzed using a multiple logistic regression model, with the findings presented in Table 3. The multivariate analysis revealed that the presence of depressive symptoms was significantly correlated with a lack of formal education (*p* = 0.048), lower income levels (*p* = 0.003), living alone (*p* = 0.025), and the occurrence of cataracts without surgical intervention (*p* = 0.007).

**Table 3:** Multivariate analyses of the associated factors for the presence of depressive symptoms.

	Multivariate analysis		
	OR	95% CI	*p*
Age groups			
65–69 years		Reference	
≥ 70 years	1.16	0.84–1.62	0.311
Sex			
Male		Reference	
Female	1.21	0.87–1.66	0.287
Educational level			
Formal education		Reference	
No formal education	1.43	0.96–2.05	**0.048**
Monthly income			
≥ 1,000 Yuan		Reference	
< 1,000 Yuan	1.55	1.15–2.09	**0.003**
Living alone or not			
No		Reference	
Yes	1.48	1.01–1.96	**0.025**
Cataract with surgery			
Yes		Reference	
No	1.53	1.10–1.81	**0.007**

Bold values mean *p* < 0.05.

## Discussion

In this community-based survey involving Chinese adults aged 65 years and older, we found that age-related cataracts, encompassing both bilateral and unilateral forms, were significantly correlated with the presence of depressive symptoms as assessed by the HAMD after adjusting for a comprehensive array of potential confounding variables. This correlation was determined to be independent of socioeconomic status, lifestyle factors, and presenting visual acuity. Notably, the relationship between age-related cataracts and depressive symptoms was influenced by the educational attainment of the individuals. These findings offer preliminary insights into the potential link between cataracts and depression. It is imperative for ophthalmologists to recognize the heightened risk of depression in patients with cataracts and to implement screening for depressive symptoms or refer patients for counseling within clinical settings. Furthermore, the results underscore the importance of timely cataract surgery as a means to mitigate the risk of depression among older adults.

Immediate Sequential Bilateral Cataract Surgery (ISBCS) demonstrates outcomes that are comparable to those of delayed sequential surgeries while exhibiting a low incidence of bilateral endophthalmitis. Furthermore, ISBCS has the potential to be both cost-effective and efficient (22). The current study represents a population-based investigation that directly evaluates the association between cataracts and depressive symptoms. Our study found a strong link between age-related cataracts and a higher chance of experiencing depressive symptoms, especially among those without formal education, which is consistent with earlier research findings (23). Numerous studies have indicated that visual impairment may serve as an independent risk factor for depression; however, the results across various studies have been inconsistent (6–9). These discrepancies may stem from differences in study design and the characteristics of the populations examined, including the varied instruments employed for screening depressive symptoms. It is important to note that visual acuity reflects a composite effect of various ocular disorders, and there is limited evidence regarding the specific impact of individual eye disorders on depression or depressive symptoms. Other research has demonstrated a significant reduction in depressive symptom scores following cataract surgery in older populations (10–14). Nevertheless, there is a paucity of population-based data exploring the relationship between cataracts and depression or depressive symptoms. This suggests that the relationship between cataracts and depressive symptoms may not be solely attributable to poor visual acuity but could also involve other vision-related factors such as halos, contrast sensitivity, and light adaptation. In addition, emotional factors, including apprehension regarding surgical procedures and frustration stemming from limitations in daily activities, may have further contributed to the observed effects on depressive symptoms in this study.

It is recognized that depression is associated with many diseases, such as digestive disorders (24), while the relationship between cataracts and depressive symptoms remains unclear. The biological mechanisms that elucidate the associations between cataracts and depressive symptoms remain inadequately understood and require further investigation. It is well-documented that age-related cataracts are the predominant cause of visual impairment among the elderly population. The resultant vision loss may diminish individuals’ capacity to engage in activities of daily living and hinder their ability to communicate. However, our findings suggest that this association is independent of the vision loss attributable to cataracts. One possible explanation for this observation is that both age-related cataracts and depression may share common risk factors, such as oxidative stress (25). Meanwhile, previous reviews indicated that pathophysiological conditions, such as inflammation and neurodegeneration, could play a role in both depression and specific eye disorders. In addition, physical symptoms and changes in bodily functions, such as disturbances in circadian rhythms caused by eye diseases, may also affect the mood of patients (26). Alternatively, individuals experiencing depression may be less inclined to seek treatment for cataracts compared to those with stable mental health. In addition, our study revealed that the correlation between age-related cataracts and depressive symptoms was more pronounced among individuals lacking formal education. Adults with varying educational backgrounds may confront distinct psychosocial challenges due to differences in lifestyle, responsibilities, or circumstances. Furthermore, perceived financial barriers to accessing eye care services and a lack of awareness regarding the potential benefits of cataract surgery may deter many individuals with limited education from pursuing medical assistance. The interaction effect identified in this study indicates a complex relationship among ocular disorders, socioeconomic status as indicated by educational attainment, and mental health, which warrants further exploration.

The findings of our study carry significant public health implications that warrant attention. Mental health issues among the elderly population represent a critical concern in China and other nations, often remaining under-identified and inadequately addressed. Given that cataracts can be effectively treated through surgical intervention, it is advisable to allocate efforts and resources toward cataract surgery programs for older adults experiencing depression, particularly in rural regions where educational levels may be low. In addition, there is a need for further randomized controlled trials to investigate the effects of cataract surgery on depressive symptoms within these demographics.

It is essential to acknowledge certain limitations. Cultural factors may significantly influence mental health, suggesting that findings from the Chinese population may not be readily applicable to other ethnic groups due to substantial cultural disparities. Moreover, despite controlling for a broad range of confounding variables in our multivariate analysis, the possibility of residual confounding cannot be dismissed. The cross-sectional design of the study also limits our ability to ascertain whether age-related cataracts precede depressive symptoms. It remains plausible that factors associated with depression could lead to environmental exposures contributing to the development of cataracts.

In summary, our research identifies a significant association between age-related cataracts and depressive symptoms among older Chinese adults, particularly those with lower educational attainment. Although the direction of causality remains ambiguous in this cross-sectional analysis, our findings illuminate the intricate relationship between aging, vision impairment, cataracts, and depression, suggesting a potential role for cataract surgery in enhancing mental health outcomes among the elderly.

## Data Availability

The original contributions presented in the study are included in the article/supplementary material, further inquiries can be directed to the corresponding author.
